# Integration of Newer Genomic Technologies into Clinical Cytogenetics Laboratories

**DOI:** 10.3390/genes16060688

**Published:** 2025-06-04

**Authors:** Patrick R. Gonzales

**Affiliations:** Department of Pathology and Laboratory Medicine, University of Kansas Medical Center, Kansas City, KS 66160, USA; pgonzales@kumc.edu; Tel.: +1-(913)-574-0288

**Keywords:** cytogenetics, fluorescence in situ hybridization (FISH), chromosomal microarray, optical genome mapping, genomic proximity mapping

## Abstract

Over the past several decades, clinical cytogenetics has branched out from the use of light microscopy and examination of banded chromosomes to embrace multiple newer techniques, including fluorescence in situ hybridization (FISH), multiple generations of microarray designs, as well as the newest technologies, namely, optical genome mapping (OGM) and genomic proximity mapping (GPM). While these newer technologies have had an increasingly molecular genetic focus over time, they are still rooted in the field of cytogenetics, the genetics of the single cell. This review provides a brief overview of the earliest, as well as the most recent, techniques available to clinical cytogenetics laboratories for both constitutional and neoplastic testing and discusses some advantages and disadvantages of each.

## 1. Introduction

For several decades, clinical cytogenetics has used light microscopy and examination of banded chromosomes to determine numerical and structural abnormalities in constitutional and neoplastic samples. Although cytogenetics started as analysis of the “single cell”, it has since branched out to embrace multiple newer cytogenetic and cytogenomic techniques, including fluorescence in situ hybridization (FISH), multiple generations of microarray designs, as well as the newest technologies, namely, optical genome mapping (OGM) and genomic proximity mapping (GPM). These innovations have resulted in increasingly higher resolution, progressing from the 3~5 Mb of a single cytogenetic band to a few kb from probes on a SNP array [1,2] and to a few kb involved in structural rearrangements as seen by OGM and GPM [3]. This review provides a brief overview of the earliest, as well as the most recent, techniques available to clinical cytogenetics laboratories for both constitutional and neoplastic testing and discusses some advantages and disadvantages of each.

## 2. Classical Cytogenetics

Since the 1970s, classical cytogenetics has been used to detect numerical and structural chromosomal abnormalities in patients with constitutional (germline) and neoplastic genetic diseases. The implementation of G-banding with light microscopy allowed for cytogeneticists to determine the identity of chromosomes involved in such numerical abnormalities as trisomy 21 in Down syndrome [4], and structural variants (SV) including the translocation t(9;22)(q34;q11.2) in chronic myeloid leukemia (CML) and B-cell acute lymphoblastic leukemia (B-ALL) [5].

**Figure 1:**
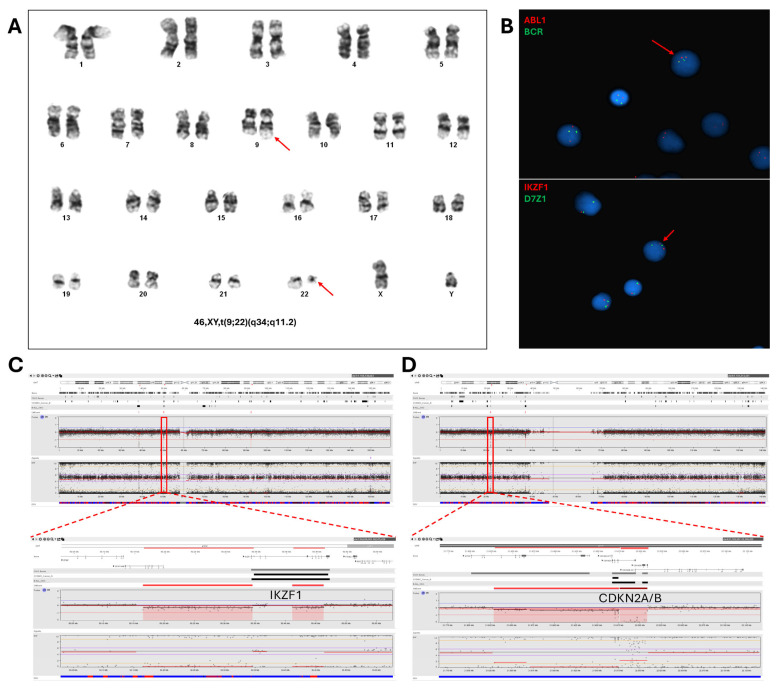
Comparison of G-banding, FISH, and SNP array (CMA) results from a patient with B-cell acute lymphoblastic leukemia (B-ALL). (**A**) Karyogram showing the classic t(9;22) rearrangement. (**B**) Interphase FISH showing *BCR*::*ABL1* positivity with dual-color/dual-fusion FISH signals in upper panel and, in lower panel showing *IKZF1* FISH with partial loss of *IKZF1*, with one diminished red signal. (**C**) CMA showing two adjacent *IKZF1* deletions, which is consistent with the diminished red signals observed by FISH. (**D**) CMA showing *CDKN2A* and *CDKN2B* with heterozygous (Left) and homozygous deletions (Right). Note lack of copy-number abnormality at 9q34 with balanced t(9;22) rearrangement. These results are consistent with B-ALL, *IKZF1*(+) and poor prognostic risk.

Improvements in culturing and harvesting techniques, including the use of ethidium bromide or other DNA intercalators, allowed for high-resolution chromosome analysis to facilitate detection of smaller deletions and/or duplications in the genome of patients presenting with such constitutional abnormalities as dysmorphic features, intellectual disability, autism spectrum disorder, failure to thrive, and reproductive issues [6,7]. While the G-banded karyotype is historically considered to be the original whole-genome analysis, its resolution is limited by variations in quality of chromosome morphology and staining to an effective limit of 5~10 Mb (Figure 1; Table 1) [1]. 

## 3. FISH

Fluorescence in situ hybridization (FISH) was implemented in clinical cytogenetics laboratories starting in the 1990s and allowed for targeted analysis of specific chromosomal regions that may be involved in constitutional or neoplastic disease [9]. The ability of FISH to use non-dividing cells has proven to be a major improvement to clinical testing [10]. Various strategies include enumeration probes to determine copy number of specific chromosomes (e.g., X, Y, 13, 18, and 21 in pre-natal testing), break-apart probes (BAP) to determine if a gene of interest (e.g., *MYC*) is rearranged in lymphoma, and dual-color/dual-fusion probes (DC/DF, e.g., *BCR*::*ABL1*) to detect recurrent abnormalities like the t(9;22) in CML and B-ALL. Although FISH can detect balanced rearrangements and cryptic deletions, because FISH is a targeted method, laboratories must choose which probe(s) to analyze for a given specimen (Figure 1) [11]. One enhancement to targeted FISH is the use of inverted-DAPI applications in digital imaging systems to visualize locations of fluorescent probes on metaphase chromosomes, which is helpful to investigate regions that may be involved in subtle chromosomal rearrangements. Another exception to the targeted nature of FISH is multicolor FISH, also known as M-FISH (Multiplex FISH) or SKY (Spectral Karyotyping) FISH, which uses whole-chromosome fluorescent probes on metaphase spreads [12,13]. Although M-FISH/SKY allows for genome-wide analysis to determine complex structural rearrangements, especially in cancer, the technique requires specialized reagents and expertise, and it is not broadly used in many clinical cytogenetics laboratories [14].

## 4. Microarrays

Microarray-based techniques that broadened the field from the single cell-based approach of classical cytogenetics into cytogenomics arrived in clinical cytogenetics laboratories in the early 2000s, with the implementation of BAC (bacterial artificial chromosome)-based comparative genomic hybridization (CGH) arrays, using DNA extracted from blood, bone marrow, or tissue. These early microarray platforms had limited coverage of the genome and were hindered by technical constraints involved in the production of the arrays, with spotted BAC DNA as the probe material [15,16]. The late 2000s saw the introduction of oligonucleotide CGH arrays, with improvements in probe coverage of the genome, increased reproducibility of results and enhanced sensitivity to detect smaller deletions and duplications, including mosaic abnormalities [17,18]. Addition of single-nucleotide polymorphism (SNP) probes to existing CGH array designs resulted in hybrid CGH+SNP arrays, which allowed for the detection of large regions of homozygosity (ROH) in constitutional samples, and copy-neutral loss of heterozygosity (CN-LOH) in neoplastic samples. Such improved sensitivities to detect constitutional abnormalities over conventional cytogenetic testing led the American College of Medical Genetics and Genomics (ACMG) to designate chromosomal microarray analysis (CMA) as first-tier testing for individuals with developmental disabilities or congenital abnormalities [19,20].

The 2010s saw the introduction of true SNP-based microarrays into clinical service, with vastly increased numbers of probes and enhanced coverage of the genome [18,21,22,23]. These SNP arrays had the advantages of robust sensitivity to detect smaller ROH, CN-LOH, and lower-level mosaic deletions and duplications, as well as abnormalities in ploidy (e.g., triploidy/tetraploidy) [2]. The ability of SNP arrays to detect these abnormalities was ideal for neoplastic testing, with the abnormalities typically mosaic, and with increased incidence of homozygosity via CN-LOH [21]. Although CMA is a comprehensive genome-wide method, it cannot detect balanced chromosomal rearrangements like translocations, inversions and insertions, or very low-level mosaicism at less than 15~20% abnormal cell population, depending on the size of the abnormality (Figure 1) [20]. While SNP arrays can detect ROH/LOH, they are not designed to detect uniparental disomy (UPD) per se, as approximately one-third of UPD events are derived from complete heterodisomy and are thus undetectable by SNP array, requiring molecular methods to fully detect and assess them for parent-of-origin imprinting [24].

## 5. Optical Genome Mapping

**Figure 2:**
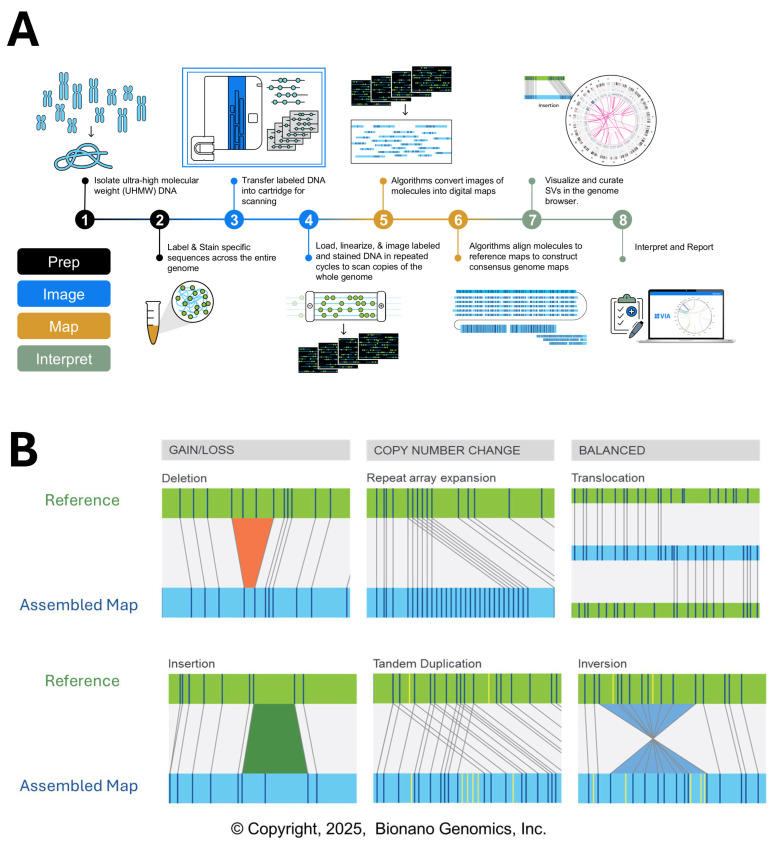
(**A**) Experimental workflow of optical genome mapping (OGM). (**B**) Identification of distinct variant classes by OGM (Figure provided courtesy of Bionano. © 2021–2025 Bionano Genomics, Inc. Used by permission. All rights reserved).

Optical genome mapping (OGM) is one of the newer technologies to enter use in some clinical cytogenetics laboratories, starting in the late 2010s, and has been successfully implemented for testing of constitutional and neoplastic samples. Through this method, ultra-high molecular weight (UHMW) DNA derived from fresh blood, bone marrow, cultured cell lines, or fresh solid tissue is labeled with a proprietary enzyme that targets the sequence recognition motif “CTTAAG” with an expected labeling frequency of 14~15 labels per 100 kb in human genomic DNA [25,26]. After counterstaining of backbone genomic DNA, the labeled UHMW DNA is applied to a flow cell (Bionano Genomics, Inc., San Diego, CA, USA) and linearized within nanochannels via electrophoresis, imaged in a scanner with a fluorescence microscope (Bionano), and converted into virtual DNA molecules that are then filtered for quality and assembled de novo into maps for comparison with a reference human genome [25,26]. With DNA molecules of up to 2 Mb in length, and with an N50 from ~130–400 kb and a map rate of 70~91%, OGM typically results in 600~900 Gb of data per sample and an average effective genomic coverage of over 300×, which is the total length of filtered (≥150 kb) and aligned molecules divided by the length of the reference genome after de novo assembly [26,27,28,29,30]. After filtering and alignment, structural variant (SV) and copy-number variant (CNV) pipelines (Bionano) are used to analyze the reads for structural and/or copy number abnormalities, respectively (Figure 2) [26].

**Figure 3:**
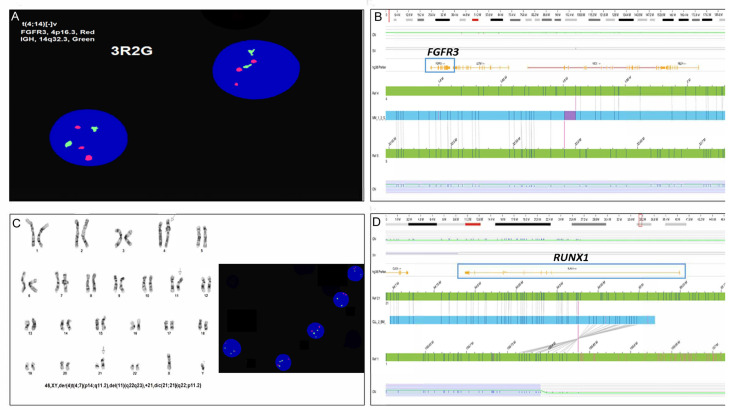
OGM corrected previously incorrect interpretations due to low resolution of karyotyping in two cases. (**A**) In a multiple myeloma case, FISH testing shows a gain of one copy of *FGFR3* on 4p [23/50 cells]; (**B**) OGM revealed that the gain observed by FISH was by a t(4;5)(p16.3; p13.3), with the breakpoint at 4p16.3 that overlaps the region of FISH dual-fusion probe used for detecting *FGFR3* gain. The breakpoint of the translocation did not disrupt the *FGFR3* gene. (**C**) In a CLL case, karyotyping detected dic(21;21)(q22; p11.2)[12/20], and interphase FISH with *RUNX1* probe showed gain of *RUNX1*. (**D**) OGM showed the dic(21;21) as most likely a der(21)ins(21;1)(q22.12; q?21.1q23.1)inv dup(q?21.1q23.1) with a copy number gain at the breakpoint on chromosome 1 (Adapted from [28] with Open Access).

OGM has been shown to be highly effective in detecting balanced structural abnormalities like translocations, inversions, and insertions. OGM has also fully characterized complex chromosomal rearrangements that were not readily apparent by G-banded, FISH, or CMA analysis [26,27,28,30]. OGM has been successfully used clinically for determination of complex abnormalities in multiple constitutional cases [3,27,30,31,32,33,34] and has also been effective in characterizing abnormalities of diagnostic and prognostic significance in hematological neoplasia (Figure 3; Table 1) [12,27,28,29,35,36,37,38,39,40,41,42,43,44,45,46].

OGM has several advantages over conventional cytogenetics and FISH, including the ability to forego cultured cells, provide genome-wide analysis of complex balanced and unbalanced structural variants with fusion detection, location and orientation of CNVs [29]. While detection of ROH/LOH and some UPD is possible in OGM, the lower size limit is ~25 Mb and is therefore less sensitive than SNP array with lower size limits for detection and reporting of 3~5 Mb [24,29]. Other technical limitations of OGM include the inability to detect whole-arm translocations (e.g., Robertsonian), and structural variants in repetitive or telomeric regions that have no unique genomic labels [46]. OGM is also unable to detect ploidy abnormalities [30,37]. Because of the strict sample requirements necessary for UHMW DNA, only fresh blood, bone marrow, or tissue samples (≤4 days old) that have been stabilized and stored at −20°C/−80°C are acceptable for use in the OGM assay [26,27,28]. Unlike CMA and FISH, no formalin-fixed paraffin-embedded (FFPE) tissue specimens can be processed and analyzed for OGM due to degradation of the necessarily long DNA molecules [34]. The use of archival fixed cytogenetic pellets may be feasible for OGM if the genomic DNA has not been degraded, but this has not been fully validated (Bionano). While the cost of validating and implementing OGM may be initially higher than other genomic technologies, the scalability of the assay may be advantageous for small to medium-sized cytogenetics laboratories [25,35,36]. As this technology matures, faster turnaround times are expected, but very complex abnormalities are likely to take several weeks to fully analyze [27].

## 6. Genomic Proximity Mapping

**Figure 4:**
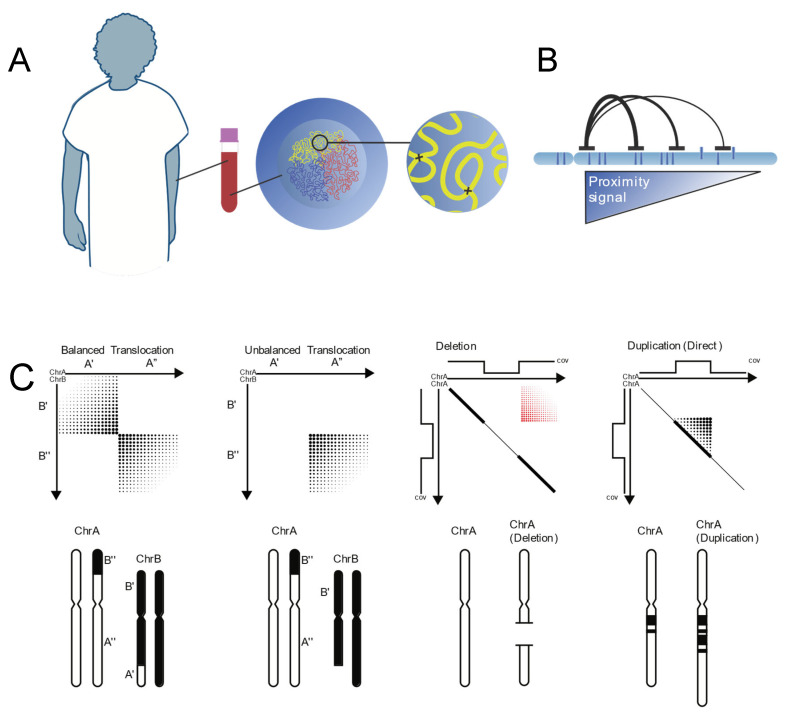
Principles of genomic proximity mapping (GPM). (**A**) Cellular samples are collected from patients and subjected to crosslinking while still intact, freezing native three-dimensional chromatin conformation in place prior to proximity ligation and library generation. (**B**) The frequency that pairs of sequences physically interact is governed primarily by their distance along the linear length of a chromosome. Using this information, the CytoTerra (Phase Genomics) variant callers can identify abnormalities in chromosome structure. (**C**) A visual guide to how classes of chromosome aberrations appear on the GPM sequence interaction matrix. Genomic coordinates are mirrored on X and Y axes while sequence interaction frequency is represented with increasing intensity on the heat map. Using a combination of interaction frequency and sequencing coverage depth, GPM can identify every major class of structural variation (Figure provided courtesy of Phase Genomics, Inc.).

Genomic proximity mapping (GPM) is the newest platform to arrive in clinical cytogenetics laboratories and is currently in the validation stages for constitutional and neoplastic testing [3,47,48,49,50]. Also known as high-throughput chromosome conformation capture (Hi-C) or proximity ligation sequencing (PLS), GPM can capture ultra-long-range genome interactions via frequency estimation of pairwise crosslinked sequence interactions to determine the structure of chromosomes and detect balanced and unbalanced chromosome rearrangements such as translocations, inversions, and insertions, as well as CNVs and CN-LOH [47]. In the GPM workflow, 2~5 × 10^5^ cells from fresh or fixed samples are suspended in buffer, chromatin in nuclei are crosslinked with formalin to preserve chromatin interaction structure, cells are bound to magnetic beads and lysed prior to restriction digestion and fill-in with biotinylated nucleotides, followed by proximity ligation, bead purification, and generation of Illumina-ready paired-end sequencing libraries, with average depth of coverage of 100× with 150~200M paired-end reads. These reads are mapped to a reference human genome and computationally processed into heatmaps showing pairwise chromosome interactions throughout the genome for determination of structural variation, with CNV and CN-LOH determination provided by sequence coverage and allele-frequency based methods (Phase Genomics, Inc., Seattle, WA, USA) (Figure 4; Table 1) [3,39,47,50].

**Figure 5:**
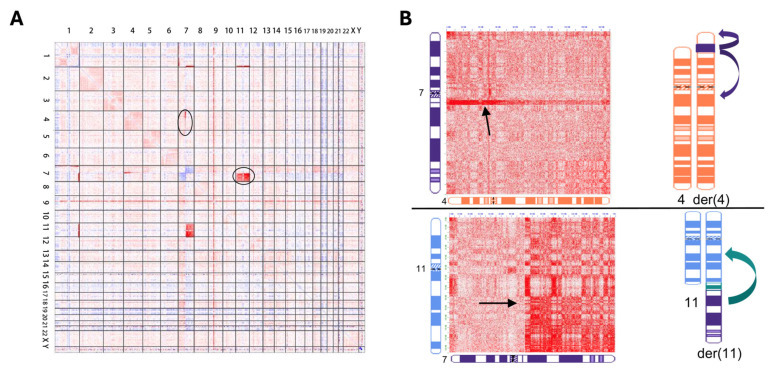
GPM results for a patient with multiple constitutional inter-chromosomal rearrangements, including ins(4;7) and t(7;11). (**A**) Genome-wide differential heatmap showing interactions between chromosomes 4 and 7, circled upper left, and chromosomes 7 and 11, circled mid-right. Red regions indicate increased interactions, with blue regions indicating reduced interactions. (**B**) Corresponding chromosome-specific heatmaps and idiograms for the 4;7 and 7;11 rearrangements. The ins(4;7) is indicated in the upper panel by increased interaction intensity between chromosome 4p and chromosome 7q in the Hi-C heatmap, with the t(7;11) in the lower panel showing increased interaction intensity between 7q and 11q. Representative idiograms are shown on the right (Adapted from [3] with Open Access).

GPM has also shown promising results in detecting complex chromosomal rearrangements in constitutional and neoplastic diseases. Two recent constitutional studies showed GPM to be comparable to OGM in characterizing highly complex translocations and insertions [3,49]. In one of these studies, a patient with intellectual disability and immunodeficiency was found to share multiple structural rearrangements with her unaffected mother, including a cytogenetically visible t(7;11). GPM showed that the patient and mother also shared a cryptic insertion of material from chromosome 7 into chromosome 4p (Figure 5), among other cryptic rearrangements. Inheritance of a rec(7) from the der(7) in the patient’s mother resulted in the abnormal phenotype [3]. Neoplastic studies using GPM with AML and solid tumors have also shown comparability to OGM and next-generation sequencing (NGS) testing, with the ability to use FFPE-derived DNA as an advantage of GPM technology [38,48,50].

GPM is unique among genomic technologies in the ability to detect and distinguish Robertsonian translocations from free trisomies via long-range three-dimensional interactions across the centromeres and along whole chromosome arms, for example, in a prenatal case with an unbalanced Robertsonian translocation: 46,XY,der(13;14)(q10;q10),+14; in another case with a balanced Robertsonian translocation: der(13;15)(q10;q10); and in a third case with a der(4;22)(p10;q10) [47]. Given the markedly increased recurrence risks of reproductive sequelae in carriers of Robertsonian translocations, the ability to detect these abnormalities via genomic methods is significant (Table 1). While no genomic methods can directly “access” heterochromatic regions of the genome because of their repetitive nature, rearrangements across heterochromatin (e.g., Robertsonian translocations) can be determined if their proximity via adjacent euchromatic regions in three-dimensional space is detected at frequencies greater than baseline [8].

The implementation of GPM in cytogenetics laboratories does not necessarily require new equipment if an affiliated molecular genetics laboratory offering NGS testing is available. The ability to generate Illumina-compatible paired-end NGS libraries to sequence on a MiSeq/NextSeq or similar device and to use dedicated software to analyze the GPM sample data (Phase Genomics, Inc.) are the requirements. Some challenges for validation and implementation of GPM in clinical cytogenetics laboratories include the non-intuitive nature of the heatmap data visualization for cytogeneticists and an associated learning curve [3,8]. Of further note is reduced sensitivity for low-level clonality, with ~91% sensitivity for CNV detection [47]. Small structural rearrangements like insertions are only detectable at 5~10× coverage and at mosaicism levels of 25~50%, with larger rearrangements detectable at 2~10× coverage and at mosaicism down to ~10% [8]. Like OGM, GPM is also unable to detect abnormalities with ploidy [51]. Additional challenges for GPM include assembly errors such as false inversion and scaffold misplacement, along with limited coverage of the linked reads to source DNA fragments [51]. Although GPM utilizes mature NGS technology with its associated costs for reagents and equipment, reaching clinical-grade accuracy for phasing structural variants will require higher coverage and orthogonal validation. As with OGM, highly complex GPM datasets will likely require several weeks to fully analyze (Table 1).

## 7. Discussion/Summary

For decades, clinical cytogenetics has used light microscopy and examination of banded chromosomes and has embraced multiple newer techniques, including fluorescence in situ hybridization (FISH), multiple generations of microarray designs, as well as the newest technologies, namely, optical genome mapping (OGM) and genomic proximity mapping (GPM). While these newer technologies have had an increasingly molecular genetic focus over time, they are still rooted in the field of cytogenetics, the genetics of the single cell. Although many cytogenetics laboratories still use the classical, as well as the most recent, techniques for both constitutional and neoplastic testing, each assay has advantages and disadvantages. No single cytogenetic or molecular assay can give all the answers [52]. The integration of data from multiple techniques is critical for success in the clinical cytogenetics laboratory.

## Figures and Tables

**Table 1:** Comparison of cytogenetic and cytogenomic technologies (Adapted from [3,8] with Open Access and permission, respectively). * See details in the manuscript.

	Karyotype	FISH	CMA	OGM	GPM
**Region**	Genome-Wide	Targeted *	Genome-Wide	Genome-Wide	Genome-Wide
**Resolution**	5–10 Mb	100 kb *	40 kb	5–500 kb	5–50 kb
**Detection of CNV**	Yes	Yes	Yes	Yes	Yes
**Detection of Ploidy**	Yes	Yes	Yes *	No	No
**Detection of Mosaicism**	Yes	Yes	Yes *	Yes *	Yes *
**Detection of LOH/ROH**	No	No	Yes	Yes *	Yes
**Detection of UPD**	No	No	Yes *	Yes *	Yes *
**Unbalanced SV**	Yes	Yes	Yes	Yes	Yes
**Balanced SV**	Yes	Yes	No	Yes	Yes
**Reconstruction of Complex CR**	Yes	No	No	Yes	Yes
**Robertsonian Translocations**	Yes	No *	No	No	Yes
**Culturing Required?**	Yes	No *	No	No	No
**Cost**	Low	Low	Low	Medium-High	Medium-High
**TAT**	Days-Weeks	Days	Days	Days-Weeks	Days-Weeks
